# Supplementary material for: Exploring the impact of a life education program on the resilience of nursing students
Source: PLoS One. 2025 Apr 29;20(4):e0322793. doi: 10.1371/journal.pone.0322793 (PMC12040196; doi:10.1371/journal.pone.0322793)
Supplement: Attachment — Submitted filename: Comments to Editor.pdf [file pone.0322793.s002.pdf]

Dear Editor,

1. Inadequate sample size: The study used only 87 participants (40 in the experimental group, 47 in control), which is relatively small for a quantitative study aiming to demonstrate intervention effects. This limits statistical power and increases the risk of Type II errors.
2. Low reliability of the Meaning in Life Scale: The reported Cronbach's alpha values for this scale were concerningly low (0.60 pre-test and 0.59 post-test for the experimental group; 0.52 pre-test and 0.58 post-test for control group). These values are below the generally accepted threshold of 0.70, calling into question the internal consistency and reliability of this key measure.
3. Lack of randomization: The study used a quasi-experimental design with convenience sampling, where "students in classes that selected the life education program formed the experimental group." This non-random assignment introduces potential selection bias and threatens internal validity.
4. Short intervention period: The life education program consisted of only 5 weeks (10 sessions of 50 minutes each). This relatively brief intervention may not be sufficient to produce lasting changes in complex psychological constructs like resilience and attitudes toward life and death.
5. Absence of follow-up assessment: The study only measured outcomes immediately after the intervention. Without a longer-term follow-up, it's impossible to determine if the observed effects were sustained over time.
6. Potential instructor bias: The paper states that the researcher developed and implemented the intervention. This dual role could introduce bias in program delivery and potentially influence participant responses.
7. Limited description of control group conditions: The study doesn't adequately describe what, if any, activities or education the control group received during the intervention period. This makes it difficult to isolate the specific effects of the life education program.
8. Inconsistent effect sizes: While the study reports statistically significant effects, the magnitude of these effects varies considerably across outcomes. For instance, the effect on life attitudes ($B = 11.29$) is much larger than on meaning in life ($B = 4.09$) or positive psychology ($B = 4.81$), without a clear explanation for this discrepancy.
9. Lack of attention to potential confounds: The study doesn't address or control for other factors that could influence outcomes, such as concurrent coursework, personal life events, or pre-existing differences in death anxiety or exposure to death-related experiences.
10. Overreliance on self-report measures: All outcomes were assessed using self-report scales, which are susceptible to social desirability bias, especially given the non-blinded nature of the intervention.

My decision would be to recommend "**Major Revision**" for this paper. The authors should address the limitations, particularly by acknowledging the generalizability issues, discussing potential biases, and providing a more critical interpretation of their findings in light of these limitations. Additionally, they

should consider conducting further research with a larger, more diverse sample, incorporating an active control group, and including longer-term follow-up assessments to strengthen the validity and reliability of their conclusions.
