# Supplementary material for: Exploring the impact of a life education program on the resilience of nursing students
Source: PLoS One. 2025 Apr 29;20(4):e0322793. doi: 10.1371/journal.pone.0322793 (PMC12040196; doi:10.1371/journal.pone.0322793)
Supplement: Attachment — Submitted filename: walaa badawy reviewer.docx [file pone.0322793.s001.docx]

First of all, I thank you for this good work, which is an addition to the field of specialization

Here are the potential errors and observations about the article

**For the research summary**

The phrasing of the objective could be improved to be clearer and more specific, such as indicating whether the focus is on enhancing particular aspects of psychological resilience. And Missing Participant Characteristics: Information about the characteristics of the participants (such as age, gender, or educational background) is not provided, which may affect the understanding of the results. And Lack of Clarity on Outcomes: While positive effects are mentioned, there are not enough details on how these results were measured or what the percentages of change were.Unsupported Recommendations: The recommendations provided need further clarification on how to implement them and what they practically mean for educational institutions. Lack of Logical Sequence**: The sequence of information in the abstract could be improved to make it easier to read and understand.

**For the introduction:**

Lack of Research Objective Clarity**: The objective of the study should be more specific, including what the study aims to achieve specifically in the field of life education.

Lack of Logical Sequence**: The information in the introduction may not be arranged logically, making it difficult for the reader to follow the main idea.

Lack of Literature Review**: Although some studies are mentioned, the introduction lacks a comprehensive review of related literature that could enhance the research context.

Insufficient Definition of Key Terms**: Some terms, such as "psychological resilience" and "life education," are not adequately defined, which may cause confusion for the reader.

Insufficient Clarification of Research Gaps**: There is not enough emphasis on the gaps in previous research that this study aims to address.

Lack of Emphasis on Research Importance**: It should be clarified why this study is important and what potential benefits may arise from its results.

Lack of Clarity in Style**: Some sentences may be complex or lengthy, making it difficult to understand the intended meaning.

Improving the introduction by addressing these points can enhance its effectiveness and increase the clarity of the research.

1. **Research Design**

A sample of 87 students may be too small to generalize the results to all nursing students.

- **Sampling Method**: Relying on convenience sampling may lead to bias in the results, as the students who choose to participate may have specific characteristics that differ from those who do not.

2. **Measurement Tools

- **Reliability of Tools**: It is important to clarify how the reliability of the tools used was measured. Although some values are mentioned, there should be more details on how these tools were tested.

- **Cultural Adaptation**: The tools used may need to be adapted to fit the local cultural context, especially if they are derived from foreign studies.

3. **Statistical Analysis

- **Lack of Clarity in Procedures**: The use of Generalized Estimating Equations (GEE) should be explained more clearly, particularly regarding continuous data and outcomes.

- **Analysis of Confounding Variables**: There is no mention of whether there were other variables that could affect the results that were not controlled for.

4. **Discussion and Conclusions**

- **Interpretation of Results**: The discussion should include a deeper analysis of the results, including comparisons with previous studies.

- **Unsupported Recommendations**: Some recommendations, such as integrating life education into the curriculum, need more evidence to support them.

5. **Ethical Considerations**

- **Ethical Conduct**: More details should be included about how ethical issues were addressed, such as how confidentiality and informed consent were ensured.

6. **Inaccurate Use of Terminology**

- **Repetitive Terminology**: Some terms, such as "meaning in life" and "positive psychology," are used repeatedly without providing new definitions or clarifications.

7. **Organization and Structure**

- **Content Organization**: There may be a need to improve the organization of the article, as paragraphs could be clarified better to allow ideas to flow logically.

Addressing these points could enhance the overall quality and credibility of the research.
