# Supplementary material for: Exploring the impact of a life education program on the resilience of nursing students
Source: PLoS One. 2025 Apr 29;20(4):e0322793. doi: 10.1371/journal.pone.0322793 (PMC12040196; doi:10.1371/journal.pone.0322793)
Supplement: Attachment — Submitted filename: Response to Reviewers-20241008.docx [file pone.0322793.s004.docx]

Manuscript Number: PONE-D-24-37761

Title: "Exploring the Impact of a Life Education Program on the Resilience of Nursing Students"

Editor and Reviewer comments:

Reviewer #1:

**First of all, I thank you for this good work, which is an addition to the field of specialization. Here are the potential errors and observations about the article:**

**For the research summary:**

1. **Phrasing of the objective** could be improved to be clearer and more specific, such as indicating whether the focus is on enhancing particular aspects of psychological resilience.

=> Thank you for your suggestion. We have revised the objective in the abstract to explicitly state that the focus of the study is on enhancing specific aspects of psychological resilience, namely meaning in life, life attitudes, and positive psychology. This provides greater clarity on the study's aims.

2. **Missing Participant Characteristics**: Information about the characteristics of the participants (such as age, gender, or educational background) is not provided, which may affect the understanding of the results.

=> We appreciate your comment. In the revised abstract, we have included key demographic information about the participants. The study involved 87 nursing students aged 20 to 25 years, with 85% of them being female. This additional information addresses your concern and provides context for the results.

3. **Lack of Clarity on Outcomes**: While positive effects are mentioned, there are not enough details on how these results were measured or what the percentages of change were.

=> We have updated the abstract to include specific details on the outcomes. The results now indicate significant interaction effects on meaning in life (B = 4.09, p < 0.001), life attitudes (B = 11.29, p < 0.001), and positive psychology (B = 4.81, p = 0.009), as well as the use of paired t-tests to confirm within-group changes. This ensures the outcomes are clearly presented.

4. **Unsupported Recommendations**: The recommendations provided need further clarification on how to implement them and what they practically mean for educational institutions.

=>We have revised the recommendations in the abstract to provide more practical implementation details. We now recommend integrating life education into nursing curricula and providing teacher training to ensure effective course delivery. We also suggest collaborating with mental health organizations to offer additional support for students, thus addressing the practical implications for educational institutions.

5. **Lack of Logical Sequence**: The sequence of information in the abstract could be improved to make it easier to read and understand.

=>We have reorganized the abstract to follow a clearer and more logical structure. It now flows from the study's objective, through the methodology and results, to the practical recommendations. This should improve readability and meet the requirements for a well-organized abstract.

**For the introduction:**

1. **Lack of Research Objective Clarity**: The objective of the study should be more specific, including what the study aims to achieve specifically in the field of life education.

=>We appreciate the reviewer’s feedback. In the revised introduction, we have made the research objective clearer by explicitly stating that the study aims to investigate the effects of life education programs on nursing students’ psychological resilience, with a specific focus on enhancing meaning in life, attitudes toward life, and positive psychology. This revision provides greater specificity regarding the goals of the study within the field of life education.

2. **Lack of Logical Sequence**: The information in the introduction may not be arranged logically, making it difficult for the reader to follow the main idea.

=>Thank you for pointing this out. We have restructured the introduction to follow a more logical sequence. The revised version first introduces the general concept of life education, followed by an explanation of its relevance to nursing students. We then systematically introduce the three key components of psychological resilience—meaning in life, attitudes toward life, and positive psychology—before discussing the gaps in existing research and the importance of this study. This restructuring enhances the overall flow and coherence of the introduction.

3. **Lack of Literature Review**: Although some studies are mentioned, the introduction lacks a comprehensive review of related literature that could enhance the research context.

=>We have expanded the literature review section to include a broader range of studies related to life education, psychological resilience, and nursing education. The revised introduction now includes references to research conducted in South Korea, China, and other countries that highlight the significance of life education in shaping nursing students' attitudes toward life, mental health, and professional competence. These additions provide a more comprehensive research context, aligning the study with relevant international findings.

4. **Insufficient Definition of Key Terms**: Some terms, such as "psychological resilience" and "life education," are not adequately defined, which may cause confusion for the reader.

=>We agree with the reviewer’s comment. In the revised introduction, we have clearly defined key terms, including "psychological resilience," "meaning in life," "attitudes toward life," and "positive psychology." Each term is explained in relation to its significance within the study and supported by relevant literature. These definitions help clarify the central concepts of the research and ensure a better understanding for the reader.

5. **Insufficient Clarification of Research Gaps**: There is not enough emphasis on the gaps in previous research that this study aims to address.

=> Thank you for this valuable suggestion. We have revised the introduction to explicitly address the research gaps that this study aims to fill. We note that few intervention studies have been conducted in Taiwan focusing on life education for nursing students, particularly in the areas of psychological resilience, meaning in life, attitudes toward life, and positive psychology. By emphasizing these gaps, we clarify the unique contribution of this study to the existing body of literature.

6. **Lack of Emphasis on Research Importance**: It should be clarified why this study is important and what potential benefits may arise from its results.

=>We appreciate the reviewer’s insight. In the revised introduction, we have emphasized the importance of this study in two main aspects: (1) the theoretical contribution to the understanding of life education in nursing, particularly in how it enhances psychological resilience; and (2) the practical implications for curriculum development in nursing education, which could provide students with the necessary tools to cope with the psychological demands of end-of-life care. These clarifications underscore the potential benefits of the study for both academic and practical applications.

7. **Lack of Clarity in Style**: Some sentences may be complex or lengthy, making it difficult to understand the intended meaning.

=> We have carefully reviewed the language and structure of the introduction to improve clarity and conciseness. Sentences have been simplified where necessary, and complex structures have been revised to ensure the text is easily understandable while maintaining academic rigor. These revisions enhance the overall readability of the introduction.

**Improving the introduction by addressing these points can enhance its effectiveness and increase the clarity of the research.**

**1. Research Design:**

- A sample of 87 students may be too small to generalize the results to all nursing students.
- => Thank you for your valuable feedback. We acknowledge that a sample size of 87 students may limit the generalizability of our findings to the broader population of nursing students. In response to this concern, we have emphasized this limitation in our revised manuscript and recommend that future studies aim for larger sample sizes to enhance the robustness and applicability of the results. By increasing the sample size, we hope to better represent the diverse experiences of nursing students and improve the overall validity of our conclusions.
  - **Sampling Method**: Relying on convenience sampling may lead to bias in the results, as the students who choose to participate may have specific characteristics that differ from those who do not.
  - =>Thank you for your insightful comment. We recognize that the use of convenience sampling can introduce bias, as participants who opt to engage in the study may possess distinct characteristics that differ from those who do not participate. In our revised manuscript, we have acknowledged this limitation and emphasized the need for future research to employ more robust sampling methods, such as randomized or stratified sampling, to enhance the representativeness of the findings and mitigate potential biases.

**2. Measurement Tools:**

- **Reliability of Tools**: It is important to clarify how the reliability of the tools used was measured. Although some values are mentioned, there should be more details on how these tools were tested.
- =>Thank you for pointing this out. We have clarified the reliability of the tools used in the study. Specifically, we employed Cronbach’s α to assess internal consistency and test-retest reliability over a two-week period for all scales used. These details have been elaborated in the methodology section to provide a clearer understanding of the reliability testing process.
- **Cultural Adaptation**: The tools used may need to be adapted to fit the local cultural context, especially if they are derived from foreign studies.
- =>Thank you for highlighting this point. We would like to clarify that while the Purpose in Life Test (PIL) and Life Attitude Scale (LAS) were initially developed by foreign researchers, they have been translated and validated by local scholars specifically for use in Taiwan. Additionally, the Positive Mental Health Scale was originally developed within Taiwan, further ensuring its cultural relevance. We have included this information in the revised manuscript to enhance clarity regarding the cultural adaptation of the assessment tools.

**3. Statistical Analysis:**

- **Lack of Clarity in Procedures**: The use of Generalized Estimating Equations (GEE) should be explained more clearly, particularly regarding continuous data and outcomes.
- => Thank you for your constructive feedback. We have revised the statistical analysis section to provide a clearer explanation of the use of Generalized Estimating Equations (GEE). GEE was selected as it allows for the analysis of repeated measurements over time and helps assess both within-group and between-group effects for continuous outcomes, such as meaning in life, life attitude, and positive psychology. The choice of GEE was appropriate given the nature of our data and the repeated measures design of the study.
- **Analysis of Confounding Variables**: There is no mention of whether there were other variables that could affect the results that were not controlled for.
- =>We acknowledge the importance of considering potential confounding variables. While this study focused on controlling for group and time, other factors were not explicitly controlled. In the Future Research section, we have recommended that future studies consider including a more comprehensive set of baseline characteristics to account for potential confounding variables, thereby strengthening the validity of the findings.

**4. Discussion and Conclusions:**

- **Interpretation of Results**: The discussion should include a deeper analysis of the results, including comparisons with previous studies.
- => Thank you for your valuable suggestion. We have expanded the discussion section to provide a more in-depth analysis of the results, including comparisons with previous studies. Specifically, we have drawn upon existing literature to further contextualize our findings on the impact of life education on psychological resilience. For example, the significant improvement in meaning in life, life attitudes, and positive psychology observed in our study aligns with previous studies from Brazil and China that highlight the role of life education in enhancing students' psychological and emotional well-being. By integrating these comparisons, we aim to provide a more comprehensive understanding of how our results fit within the broader context of life education research.
- **Unsupported Recommendations**: Some recommendations, such as integrating life education into the curriculum, need more evidence to support them.
- => Thank you for pointing this out. We agree that more evidence is needed to support the recommendation of integrating life education into the curriculum. In response, we have further emphasized the positive outcomes from our study, particularly in relation to improvements in students' meaning in life, life attitudes, and positive psychology. These findings, in conjunction with supporting literature, provide preliminary evidence for the integration of life education into nursing curricula. However, we acknowledge that further research is required to explore the long-term effectiveness of these interventions across diverse student populations and educational contexts. We have recommended this in the conclusion section as an area for future investigation.

**5. Ethical Considerations:**

- **Ethical Conduct**: More details should be included about how ethical issues were addressed, such as how confidentiality and informed consent were ensured.
- => Thank you for the reviewer’s valuable suggestion. We have now added more details to clarify how ethical issues were addressed in the study. Specifically, we have elaborated on the procedures for obtaining informed consent from all participants, ensuring confidentiality through the anonymization of the questionnaires, and securely storing data with restricted access to the research team. Additionally, participants were fully informed of their rights, including their right to withdraw from the study at any time. We have ensured that the study adhered to the ethical guidelines and regulations of the country where it was conducted. These additions aim to provide a clearer understanding of the ethical considerations implemented in the study.

**6. Inaccurate Use of Terminology:**

- **Repetitive Terminology**: Some terms, such as "meaning in life" and "positive psychology," are used repeatedly without providing new definitions or clarifications.
- =>We have restructured the Introduction to ensure that terms like "meaning in life" and "positive psychology" are defined early in the text and are not repeated without purpose. The definitions are now clear and only reiterated when necessary to support the discussion.

**7. Organization and Structure:**

- **Content Organization**: There may be a need to improve the organization of the article, as paragraphs could be clarified better to allow ideas to flow logically.
- => Thank you for the feedback regarding organization and structure. We have revised the Introduction and Discussion sections to improve clarity and ensure a logical flow of ideas. Key terms are now clearly defined, and transitions between paragraphs are smoother, enhancing readability throughout the manuscript.
- **Addressing these points could enhance the overall quality and credibility of the research.**
- => We agree that addressing these points will enhance the overall quality and credibility of the research. By refining the methodology, mitigating potential biases, and expanding the scope of future studies, we aim to ensure a more robust and reliable foundation for our findings. We are committed to making these improvements and believe they will significantly strengthen the study's impact.

Reviewer #2:

1. **Inadequate sample size**: The study used only 87 participants (40 in the experimental group, 47 in control), which is relatively small for a quantitative study aiming to demonstrate intervention effects. This limits statistical power and increases the risk of Type II errors.

=> Thank you for your feedback. We have addressed this concern in the second point of our research recommendations, where we acknowledge the sample size limitations and suggest increasing the sample size in future studies to enhance the robustness and generalizability of the findings.

2. **Low reliability of the Meaning in Life Scale**: The reported Cronbach's alpha values for this scale were concerningly low (0.60 pre-test and 0.59 post-test for the experimental group; 0.52 pre-test and 0.58 post-test for the control group). These values are below the generally accepted threshold of 0.70, calling into question the internal consistency and reliability of this key measure.

**=>** Thank you for pointing this out. While the Cronbach’s α values for the Meaning in Life Scale (0.60 pre-test and 0.59 post-test for the experimental group; 0.52 pre-test and 0.58 post-test for the control group) fall below the commonly accepted threshold of 0.70, this is discussed in the Reliability Analysis of Research Tools section.

Cronbach’s α is influenced by the number of items, and the 20-item Meaning in Life Scale likely contributed to the lower values. In contrast, the Life Attitude Scale and Positive Mental Health Scale, with 24 and 25 items respectively, had higher α values. More importantly, the test-retest reliability for the Meaning in Life Scale was strong (γ = 0.91 for the experimental group and γ = 0.76 for the control group), showing excellent temporal stability.

Thus, despite the lower α values, the high test-retest reliability and strong stability over time affirm that the Meaning in Life Scale remains a reliable measure for this study.

3. **Lack of randomization**: The study used a quasi-experimental design with convenience sampling, where "students in classes that selected the life education program formed the experimental group." This non-random assignment introduces potential selection bias and threatens internal validity.

=>Thank you for raising this concern. We acknowledge the limitation regarding the lack of randomization due to the use of a quasi-experimental design with convenience sampling. As mentioned in the Research Recommendations section, this sampling method may introduce selection bias and limit the generalizability of the findings. To address this, we recommend that future studies employ randomized or stratified random sampling to improve the internal validity and representativeness of the sample. This will help reduce potential bias and enhance the robustness of the results.

4. **Short intervention period**: The life education program consisted of only 5 weeks (10 sessions of 50 minutes each). This relatively brief intervention may not be sufficient to produce lasting changes in complex psychological constructs like resilience and attitudes toward life and death.

=> Thank you for your valuable suggestion. The original intervention duration of five weeks was designed based on recommendations from the literature, which suggested similar formats for educational interventions targeting nursing students. However, we fully agree that extending the intervention period may lead to more sustainable and lasting changes in psychological resilience. We have added this point to our Research Recommendations and Limitations section, proposing that future studies extend the intervention duration to allow students more time to internalize the concepts and further enhance the program's long-term effects.

5. **Absence of follow-up assessment**: The study only measured outcomes immediately after the intervention. Without a longer-term follow-up, it's impossible to determine if the observed effects were sustained over time.

=> Thank you for your insightful comment regarding the absence of follow-up assessment. We acknowledge the importance of evaluating the long-term sustainability of the observed effects. In response to this concern, we have addressed it in Research Recommendation 6, where we propose that future studies adopt longitudinal designs to assess whether the improvements in meaning in life, life attitudes, and positive psychology persist over time. We also recommend extending the follow-up period to ensure that the life education program’s benefits are sustained in the long term.

6. **Potential instructor bias**: The paper states that the researcher developed and implemented the intervention. This dual role could introduce bias in program delivery and potentially influence participant responses.

=> Thank you for your comment. We have addressed potential instructor bias in several sections of the manuscript:

**Intervention Design:** The program followed a structured curriculum based on established frameworks to minimize subjective influence, as outlined in the Intervention section.

**Ethical Safeguards:** In the Research Ethics section, we ensured participant independence by using anonymized, validated instruments, reducing the risk of biased responses.

**Addressing Potential Bias:** As noted in the Sampling and Potential Bias section, the researcher's dual role is acknowledged as a limitation, and future studies should involve independent facilitators and adopt randomized sampling to improve objectivity.

7. **Limited description of control group conditions**: The study doesn't adequately describe what, if any, activities or education the control group received during the intervention period. This makes it difficult to isolate the specific effects of the life education program.

=> Thank you for your feedback. We have added clarification to the Research Design section. The experimental group received the life education intervention as it was an elective course they selected, while the control group did not participate in any similar activities during the study. Since the control group did not attend classes during the intervention, potential interactions with the experimental group were minimized, ensuring the effects of the life education program were isolated.

8. **Inconsistent effect sizes**: While the study reports statistically significant effects, the magnitude of these effects varies considerably across outcomes. For instance, the effect on life attitudes (B = 11.29) is much larger than on meaning in life (B = 4.09) or positive psychology (B = 4.81), without a clear explanation for this discrepancy.

=> Thank you for the observation. The variation in effect sizes is due to differences in the scoring ranges and item counts of the scales. The Meaning in Life Scale has a larger range (20–140) compared to the Life Attitude and Positive Mental Health scales, which have smaller ranges (24–120 and 25–125, respectively). This naturally results in smaller B values for the Meaning in Life Scale. Despite this, all three measures show significant effects, indicating the intervention’s impact across the outcomes. We have clarified this in the results section.

9. **Lack of attention to potential confounds**: The study doesn't address or control for other factors that could influence outcomes, such as concurrent coursework, personal life events, or pre-existing differences in death anxiety or exposure to death-related experiences.

=> Thank you for your insightful feedback. We have addressed this concern in the fourth point of our research recommendations (Confounding Variables). While the primary variables were controlled for in the study, certain potential confounding factors—such as students' prior experiences with death, personal coping mechanisms, or concurrent coursework—were not explicitly accounted for. We recognize the importance of these variables, and future research should include more detailed baseline assessments to control for such potential confounders, providing a clearer interpretation of the intervention's effectiveness.

10. **Overreliance on self-report measures**: All outcomes were assessed using self-report scales, which are susceptible to social desirability bias, especially given the non-blinded nature of the intervention.

=> Thank you for your feedback. We acknowledge the potential for social desirability bias from self-report measures. To address this, we've highlighted in the Research Limitations that future studies should include qualitative methods, such as interviews or third-party observations, to mitigate bias and enhance understanding. Anonymity measures, as noted in the Research Ethics section, were also implemented to reduce social desirability bias in responses.

**My decision would be to recommend "Major Revision" for this paper.** The authors should address the limitations, particularly by acknowledging the generalizability issues, discussing potential biases, and providing a more critical interpretation of their findings in light of these limitations. Additionally, they should consider conducting further research with a larger, more diverse sample, incorporating an active control group, and including longer-term follow-up assessments to strengthen the validity and reliability of their conclusions.

=> Thank you for your valuable feedback. We recognize the importance of addressing the limitations highlighted, such as generalizability, potential biases due to convenience sampling, and the researcher's dual role. These concerns have been discussed in both the Research Limitations and Research Ethics sections.

We have also proposed steps for future research, including the use of independent facilitators, random or stratified sampling, larger and more diverse samples, active control groups, and longer-term follow-up assessments to improve the validity and reliability of the findings.

We appreciate your thoughtful guidance and are committed to incorporating these revisions to strengthen the manuscript.
