# Supplementary material for: Exploring the impact of a life education program on the resilience of nursing students
Source: PLoS One. 2025 Apr 29;20(4):e0322793. doi: 10.1371/journal.pone.0322793 (PMC12040196; doi:10.1371/journal.pone.0322793)
Supplement: Attachment — Submitted filename: Response to Reviewers-20250312.docx [file pone.0322793.s005.docx]

Manuscript Number: PONE-D-24-37761

Title: "Exploring the Impact of a Life Education Program on the Resilience of Nursing Students"

Comments to the Author

1. If the authors have adequately addressed your comments raised in a previous round of review and you feel that this manuscript is now acceptable for publication, you may indicate that here to bypass the “Comments to the Author” section, enter your conflict of interest statement in the “Confidential to Editor” section, and submit your "Accept" recommendation.

Reviewer #2: (No Response)

=> We appreciate the reviewer’s time and consideration in evaluating our manuscript. Thank you for your feedback and support.

2. Is the manuscript technically sound, and do the data support the conclusions?

The manuscript must describe a technically sound piece of scientific research with data that supports the conclusions. Experiments must have been conducted rigorously, with appropriate controls, replication, and sample sizes. The conclusions must be drawn appropriately based on the data presented.

Reviewer #2: Yes

=>We appreciate the reviewer’s positive evaluation and acknowledgment that the manuscript is technically sound and that the data support the conclusions. Thank you for your thoughtful review.

3. Has the statistical analysis been performed appropriately and rigorously?

Reviewer #2: Yes

=>We appreciate the reviewer’s positive assessment of our statistical analysis. Thank you for your valuable review.

4. Have the authors made all data underlying the findings in their manuscript fully available?

The PLOS Data policy requires authors to make all data underlying the findings described in their manuscript fully available without restriction, with rare exception (please refer to the Data Availability Statement in the manuscript PDF file). The data should be provided as part of the manuscript or its supporting information, or deposited to a public repository. For example, in addition to summary statistics, the data points behind means, medians and variance measures should be available. If there are restrictions on publicly sharing data—e.g. participant privacy or use of data from a third party—those must be specified.

Reviewer #2: Yes

=>We appreciate the reviewer’s acknowledgment that all data underlying our findings have been fully made available in accordance with the journal’s data policy. Thank you for your review.

5. Is the manuscript presented in an intelligible fashion and written in standard English?

PLOS ONE does not copyedit accepted manuscripts, so the language in submitted articles must be clear, correct, and unambiguous. Any typographical or grammatical errors should be corrected at revision, so please note any specific errors here.

Reviewer #2: Yes

=>We appreciate the reviewer’s positive evaluation of the manuscript’s clarity and language quality. Thank you for your review.

6. Review Comments to the Author

Please use the space provided to explain your answers to the questions above. You may also include additional comments for the author, including concerns about dual publication, research ethics, or publication ethics. (Please upload your review as an attachment if it exceeds 20,000 characters)

=>We appreciate the reviewer’s time and thorough evaluation of our manuscript. Thank you for your thoughtful review.

Reviewer #2:

**Introduction Section (Pages 4-6)**

**The introduction presents life education broadly without defining its specific benefits for resilience in nursing students, making it challenging to identify the intervention’s distinct contributions.**

**=>** We appreciate the reviewer’s insightful feedback regarding the need to clarify the specific benefits of life education for resilience in nursing students. In response, we have revised the Introduction to explicitly define psychological resilience as the study’s primary focus and its three key components: meaning in life, attitudes toward life, and positive psychology.

To strengthen the intervention’s distinct contributions, we have added more targeted discussions on how life education enhances each component of psychological resilience, particularly in reducing death anxiety, strengthening coping mechanisms, and fostering emotional resilience in end-of-life care settings. Additionally, we have clarified the existing research gap and emphasized the study’s role in addressing it through an empirically structured intervention.

These revisions ensure that the introduction clearly delineates the relationship between life education and resilience-building in nursing students, thereby improving the interpretive depth of the study’s objectives.

**Methods Section:**

**The justification for using Generalized Estimating Equations (GEE) is insufficiently detailed, particularly regarding the management of baseline homogeneity and the rationale behind selecting GEE for repeated measures analysis.**

**The sample size calculation, though meeting minimum requirements, lacks a detailed basis for assumed effect size and power analysis parameters.**

**=>We sincerely appreciate the reviewer’s valuable feedback regarding the justification for using Generalized Estimating Equations (GEE) and the details of the sample size calculation. To address these concerns, we have made the following revisions:**

1. Justification for Using GEE: We have expanded the explanation of why GEE was chosen over other repeated measures approaches, such as repeated measures ANOVA and mixed-effects models. The revised text highlights GEE’s ability to handle correlated observations, accommodate missing data, and provide robust standard error estimates when normality assumptions are violated. Additionally, we now explicitly state that an exchangeable working correlation matrix was used to model within-subject dependencies.
2. Baseline Homogeneity Management: To improve transparency in our analytical approach, we have clarified how baseline homogeneity was assessed and managed. Specifically, pre-test values of psychological resilience variables were included as covariates in the GEE model to adjust for any potential baseline differences between the experimental and control groups. This ensures that the observed intervention effects are attributable to the life education program rather than pre-existing group differences.
3. Sample Size Calculation: We have provided a more detailed justification for our sample size determination. The revised section explicitly reports the assumed effect size (Cohen’s d = 0.65 for independent t-tests and Cohen’s dz = 0.65 for paired t-tests), power (0.80), and alpha level (0.05). Additionally, we now reference G*Power 3.1 as the statistical tool used for the calculations and include supporting literature that guided the selection of these parameters.

**Results Section (Table 2 and 3):**

**Statistical tests used for comparisons in Table 2 are not specified, and p-value formatting across tables is inconsistent, impacting the clarity of the statistical findings.**

=> We appreciate the reviewer’s comments regarding the clarity of statistical tests and p-value formatting. To address these concerns, we have revised Table 2 to explicitly state that chi-square tests (χ²) were used when all expected cell counts were ≥5, while Fisher’s exact test was applied when any expected cell count was <5. This clarification has been added to both the Methods section and the table footnotes for accuracy.

Additionally, we have standardized the p-value formatting across all tables, ensuring that all p-values are reported to three decimal places (e.g., p = 0.023), with values below 0.001 presented as p < 0.001. In Table 3, we have updated the table structure to clearly differentiate between between-group (independent sample t-tests) and within-group (paired sample t-tests) comparisons by modifying the column headings to "Between-group t-value" and "Within-group t-value (p)". This adjustment enhances clarity and ensures consistency in statistical reporting.

These revisions improve the transparency and coherence of our results presentation. We appreciate the reviewer’s valuable feedback, which has helped refine the methodological rigor of our study.

**Discussion Section (Pages 18-20):**

**There is limited integration of findings with existing literature on resilience-building in nursing education, reducing the interpretive depth of the study’s outcomes.**

**Subheadings within the Discussion are inconsistently formatted, affecting the flow and readability of the section.**

=> We appreciate the reviewer’s feedback regarding the integration of findings with existing literature on resilience-building in nursing education and the formatting of subheadings in the Discussion section.

To address this, we have explicitly linked our findings to prior research on resilience by demonstrating how life education enhances meaning in life, attitudes toward death, and positive psychology—all of which contribute to resilience in nursing students. These connections have been strengthened with relevant literature, ensuring a more cohesive interpretation of the study’s outcomes.

Additionally, we have restructured the Discussion section by adding clear subheadings, ensuring a more consistent format and logical flow. These subheadings categorize key findings, making it easier for readers to follow the discussion and understand the implications of our study.

We appreciate the reviewer’s valuable suggestions, which have helped enhance the clarity, structure, and interpretative depth of our findings.

**Technical and Formatting Issues:**

**Inconsistent p-value formatting in tables and inconsistent citation formatting detract from the manuscript’s overall clarity and presentation quality.**

=>We appreciate the reviewer’s feedback regarding p-value and citation formatting inconsistencies. To address these concerns, we have standardized p-values to three decimal places (e.g., p = 0.023), with values below 0.001 reported as p < 0.001 across all tables, figures, and text. Additionally, we have reviewed and corrected all in-text citations to ensure consistency with the journal’s formatting guidelines. The reference list has also been cross-checked for accuracy and uniformity.

We have carefully examined the formatting throughout the manuscript. However, if there are any remaining errors, we kindly ask the editorial team to specify the exact areas that require correction so that we can promptly address them. We appreciate the reviewer’s valuable input in improving the clarity and presentation of our manuscript.
